# A187 DIETARY FIBERS ELICIT GUT IMMUNE AND EPITHELIAL BARRIER MODIFYING EFFECTS IN INFLAMMATORY BOWEL DISEASES BASED ON FOOD SOURCE AND FIBER CHEMICAL FEATURES

**DOI:** 10.1093/jcag/gwac036.187

**Published:** 2023-03-07

**Authors:** M Bording-Jorgensen, F Moreau, H Gorman, R Mahmood, H Olof, A Voisin, D Coker, T -L Jeanson, W El-Matary, M Carroll, H Huynh, C Bernstein, D Santer, K Chadee, E Wine, T Vasanthan, H Armstrong

**Affiliations:** 1 University of Alberta, Edmonton; 2 University of Calgary, Calgary; 3 University of Manitoba, Winnipeg, Canada

## Abstract

**Background:**

Dietary fibers are not digested in the bowel; they are fermented by microbes, typically promoting gut health. However, IBD patients experience sensitivity to consumption of fibers. Our previous findings offered the first mechanistic evidence demonstrating that unfermented dietary β-fructans (inulin and oligofructose) can induce pro-inflammatory cytokines and altered epithelial barrier integrity in a subset of pediatric IBD colonic biopsies cultured *ex vivo*, and in the SYNERGY-1 (β-fructan) clinical study of adult remission UC patients. Fermentation of β-fructan by whole-microbiota intestinal washes from non-IBD or remission IBD patients (but not non-IBD microbes) reduced pro-inflammatory responses.

**Purpose:**

Here we aimed to expand our findings to uncover the physiologically relevant gut immune and epithelial responses to over 50 unfermented and partially fermented dietary fibers (arabinoxylans, β-glucans, β-mannans, galatooligosaccharides, inulins, oligofructoses, pectins, raffinooligosaccharides, xyloglucans) sourced from commonly consumed fruits, grains, and vegetables to better understand which foods are safe for IBD patients, and in which disease state settings.

**Method:**

Colonic biopsies cultured *ex vivo*, peripheral blood mononuclear cells (PBMCs), colonic organoids, and cell lines were incubated with individual dietary fibers or mixture of fibers extracted from commonly consumed fruits, grains, and vegetables. Epithelial barrier integrity (TEER, microscopy, FITC-dextran) and immune responses (cytokine secretion [ELISA/MSD] and expression [qPCR]) were assessed. Structural features of the different fibers (*e.g.,* degree of polymerization, phenolic/phytic content, branching, sugar content) were measured by HPLC and gas chromatography and correlated to host cell responses.

**Result(s):**

Most significantly unfermented inulin, oligofructose, and arabinoxylan induced pro-inflammatory responses, particularly in myeloid cells. Pectin and galatooligosaccharides were either non-inflammatory or anti-inflammatory depending on the food source. The epithelial barrier response to select dietary fibers correlated more significantly with the chemical properties of the fibers; longer fibers (greater degree of polymerization; *e.g.,* inulin) displayed improved barrier integrity while shorter dietary fibers with higher phenolic content displayed reduced barrier integrity. Fiber structural properties varied significantly between different fiber subtypes along with the same fiber subtype sourced from different foods.

**Conclusion(s):**

Our findings suggest that intolerance and avoidance of fibers in select IBD patients occurs in patients whose gut microbiota do not support fermentation of fibers resulting in increased presence of unfermented dietary fibers in the gut. Here we show which specific dietary fibers from specific food items can elicit gut barrier damage and inflammation in the gut dependent on fiber structural features, suggesting mechanisms underlying IBD patient avoidance of specific high-fiber foods.

**Please acknowledge all funding agencies by checking the applicable boxes below:**

Other

**Please indicate your source of funding;:**

Weston Family Foundation, MMSF, NSERC, CRC

**Disclosure of Interest:**

None Declared

